# A Low Power Sigma-Delta Modulator with Hybrid Architecture

**DOI:** 10.3390/s20185309

**Published:** 2020-09-16

**Authors:** Shengbiao An, Shuang Xia, Yue Ma, Arfan Ghani, Chan Hwang See, Raed A. Abd-Alhameed, Chuanfeng Niu, Ruixia Yang

**Affiliations:** 1 School of Electronic and Information Engineering, Hebei University of Technology, Tianjin 300401, China; anshengbiao@hebust.edu.cn (S.A.); yangrx@hebut.edu.cn (R.Y.); 2 School of Information Science and Engineering, Hebei University of Science and Technology, Shijiazhuang 050018, China; xiashuang@stu.hebust.edu.cn; 3 School of Astronomy and Space Science, University of Chinese Academy of Sciences, Beijing 100049, China; yuema@nao.cas.cn; 4 School of Computing, Electronics and Maths (Research Institute for Future Transport and Cities), Coventry University, Coventry CV15FB, UK; 5 School of Engineering and the Built Environment, Edinburgh Napier University, Edinburgh EH10 5DT, UK; C.See@napier.ac.uk; 6 Faculty of Engineering and Information, University of Bradford, Bradford BD7 1DP, UK; r.a.a.abd@bradford.ac.uk; 7 Department of Communication and Informatics Engineering, Basrah University College of Science and Technology, Basrah 614004, Iraq; 8 The 54th Research Institute of China Electronic Technology Group Corporation, Shijiazhuang 050081, China; n13171886628@163.com

**Keywords:** feedforward modulator, quantizer, SAR, Σ-Δ modulator

## Abstract

Analogue-to-digital converters (ADC) using oversampling technology and the Σ-∆ modulation mechanism are widely applied in digital audio systems. This paper presents an audio modulator with high accuracy and low power consumption by using a discrete second-order feedforward structure. A 5-bit successive approximation register (SAR) quantizer is integrated into the chip, which reduces the number of comparators and the power consumption of the quantizer compared with flash ADC-type quantizers. An analogue passive adder is used to sum the input signals and it is embedded in a SAR ADC composed of a capacitor array and a dynamic comparator which has no static power consumption. To validate the design concept, the designed modulator is developed in a 180 nm CMOS process. The peak signal to noise distortion ratio (SNDR) is calculated as 106 dB and the total power consumption of the chip is recorded as 3.654 mW at the chip supply voltage of 1.8 V. The input sine wave of 0 to 25 kHz is sampled at a sampling frequency of 3.2 Ms/s. Moreover, the results achieve a 16-bit effective number of bits (ENOB) when the amplitude of the input signal is varied between 0.15 and 1.65 V. By comparing with other modulators which were realized by a 180 nm CMOS process, the proposed architecture outperforms with lower power consumption.

## 1. Introduction

The rapid development of the Internet of Things (IoTs) demands sophisticated electronics to support the vision of smart cities [1]. High-performance analogue-to-digital converters (ADCs) are frequently used in embedded systems such as mobile phones, iPads, interactive multimedia systems and so on [2,3]. All of these devices require that the conversion chip has a high signal-to-noise ratio (SNR). There are many types of ADCs [4], and the traditional ones are pipelined, successive approximation register (SAR) and Σ-Δ, which have been developed rapidly in recent years.

A traditional pipelined ADC consists of a resistor divider, comparator, buffer and encoder. Its advantage is its high A/D conversion speed; however, it suffers from having a low resolution, high power consumption, high cost and less precision. In contrast to a pipelined ADC, a SAR ADC is comprised of a comparator, a D/A converter, a comparison register SAR, a clock generator and a control logic circuit. Its operating principles are to continuously compare the sampled input signal with a known voltage and then convert it into a binary number [5,6]. Due to the matching errors of internal components of the SAR ADC, it is widely used in medium and low speed and medium-resolution sensor networks. Although the SAR ADC [7] has power consumption efficiency, it is difficult to realize high precision due to its structural characteristics and requires additional correction circuits which increases the component overhead and power consumption of the ADC [8]. To combat with the above deficiencies, the core technologies of the sigma-delta modulator which are based on oversampling and noise shaping technologies were proposed to achieve high speed and high precision. Moreover, a sigma-delta modulator can easily obtain higher performance in low-order quantization, while the quantizer of a sigma-delta modulator usually adopts the traditional flash structure, which leads to the higher power consumption of the circuit. As the sigma-delta ADC adopts the same technologies as the sigma-delta modulator, it reduces the requirements for component matching, saves cost and it is relatively easy to realize a conversion accuracy of more than 14 bits, thus being suitable for applications in low power consumption modules [9,10].

Combining the advantages of SAR and sigma-delta ADCs, this paper proposes an architecture that combines SAR and sigma-delta ADCs. That is, the sigma-delta low power consumption, high-precision modulator based on a SAR structure quantizer [11]. An ADC of this structure can reduce the quantization noise in the required signal frequency band through noise shaping and oversampling technology, improving the analogue-to-digital converter signal to noise distortion ratio (SNDR). At the same time, due to the use of a SAR quantizer, the modulator power consumption is also reduced compared with the traditional sigma-delta modulator [12].

The structure of this paper can be summarized as follows. Section 2 provides an overview of the improved modulator based on the SAR quantizer. This section is followed by the overall circuit structure verification. In Section 4, the key circuit design of the modulator is presented, including the circuit design and optimization of the quantizer module and the improvement of the amplifier. In Section 5, the integrator circuit module analysis is described and it covers the design of the transconductance operational amplifier circuit and reference voltage source, while Section 6 discusses the pre-circuit simulation and verification. The layout of the chip is presented in Section 7 and the feasibility of this structure is verified in Section 8, followed by the conclusion in Section 9.

## 2. Improved Σ-Δ Modulator Based on SAR Quantization Structure

The quantization noise is determined by the bit number of the quantizer. The higher the bit number, the smaller the noise [13]. Compared with the single-bit quantization structure, the multi-bit quantization structure not only improves the modulator performance but also reduces the total system power consumption and improves the stability of the system. However, if the quantization bit is too high, the conversion rate will be reduced.

Choosing a five-bit quantization structure and multiple bits can also reduce the performance requirements of the integrator [14]. For a stable integrator, it adopts a second-order or cascade structure as a high-order single-loop structure. Meanwhile, to reduce the power consumption and swing requirement of the modulator, the modulator adopts a low swing circuit structure [15], and the integrator only processes the residual value between the quantization result of the quantizer and the input signal, which has a small amplitude. The noise shaping effect can be achieved by using the second-order integrator structure, and the stability of the system also remains good.

**Figure 1:**
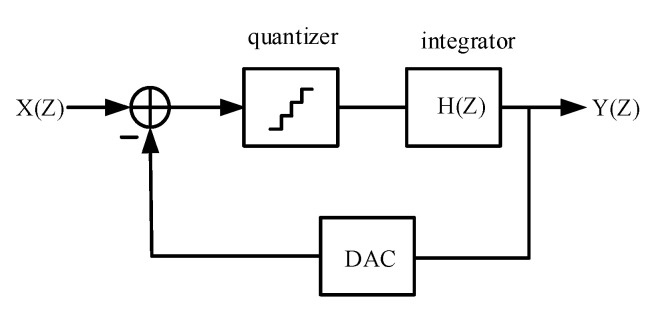
Sigma-delta analogue-to-digital converter (ADC) system block diagram.

Hence, a second-order five-bit modulator structure is proposed, as shown in Figure 1. The integrator in the modulator is formed by a discrete switched capacitor and transconductance operational amplifier, and the discrete switched capacitor makes the integrator insensitive to jitter caused by the clock [16]. Since the swing of the integrator is affected by the swing of the operational amplifier, the transconductance operational amplifier generally adopts a sleeve structure. At the same time, the active adder adds the feedforward result of the integrator, and the input signal is replaced by the passive adder, which further reduces the power consumption of the system [17].

In order to realize a low power consumption circuit, there are many existing structures and schemes for a Σ-Δ modulator module such as [18], where a Σ-Δ modulator based on the SAR quantization structure is reported. It adopts a second-order integrator with a 4-bit quantizer architecture and conducts post-simulation on the circuit behavior, the circuit itself and at the layout level, which verifies the feasibility of the scheme.

Figure 1 shows a sigma-delta ADC system framework which consists of a reusable 5-bit SAR ADC and two integrators. The input signal X(Z) first enters the quantizer for five-bit quantization. The quantization result of each bit is fed back to the input signal port through the D/A converter, and subtraction is performed with it. Therefore, the voltage processed by the quantizer is the difference between the quantization result and the input signal, and the power consumption is lower than that of the comparator. After five-bit quantization, the difference between the final quantization result and the input signal is sent to the integrator. The integrator only deals with the quantization error, and the integrated result Y(Z) is sent back and added in the next quantizer sampling to obtain higher conversion accuracy.

In a traditional feedforward modulator, an amplifier is required to form an active analogue adder [19] at the ADC input node, which increases the power consumption of the modulator. In this paper, a multi-bit feedforward [20] ADC without an active analogue adder is adopted to overcome the power issue. The passive adder embedded in the SAR ADC is implemented by using a separate capacitor array [21] and a dynamic comparator [22]. The integrator is realized by a ring amplifier without a static current. The capacitor array of the SAR ADC samples the input signal, and the capacitor C_S_ samples the integrator output. After this sampling operation, the SAR ADC quantizes the sampled signal in a binary search mode and outputs it through the Digital to Analogue Converter (DAC)Finally, a residual voltage V_RES_ is generated on the top plate of the capacitor array.

(1) $$V_{\mathrm{RES}}\left(z\right)=V_{\mathrm{IN}}\left(z\right)-V_{\mathrm{DAC}\_\mathrm{OUT}}\left(z\right)$$

where V_IN_ is the input sample signal, and V_DAC_OUT_ is the output voltage of the DAC. The residual voltage is then processed by a two-stage integrator in the integration phase of the ADC. Meanwhile, the digital output of the current sample V_DAC_OUT_ (k) can be expressed as

(2) $$V_{\mathrm{DAC}\_\mathrm{OUT}}\left(z\right)=V_{\mathrm{IN}}\left(z\right)+V_{\mathrm{RES}}*H(z)+Q\left(z\right)$$

where H(z) is the transfer function of the integrator, and Q(z) represents the quantization noise and comparator noise of the ADC. Substituting VRES of Equation (1) into Equation (2), the following system transfer function (3) is obtained.

(3) $$V_{\mathrm{DAC}\_\mathrm{OUT}}\left(z\right)=V_{\mathrm{IN}}\left(z\right)+1/\left(1+H\left(z\right)\right)Q\left(z\right)$$

The signal transfer function and noise transfer function can be obtained from Equation (3), as shown in Equations (4) and (5).

(4) $$\mathrm{STF}\left(z\right)=z^{-2}$$

(5) $$\mathrm{NTF}\left(z\right)={\left(1-z^{-1}\right)}^{2}$$

where STF is a signal transmission function, and NTF is a noise transmission function. It can be seen from Equation (5) that an NTF is a high-pass function, and the system suppresses the noise at low frequency, thus achieving a higher signal-to-noise ratio in the bandwidth.

In the proposed architecture, the SAR quantizer is not only used to realize the quantization of the modulator but is also used to realize the summation of the input signal and feedback signal in the traditional modulator. In this way, the sampling capacitance of the first integrator in the modulator can be multiplexed with the capacitance of the SAR quantizer, thus reducing the power consumption and area of the modulator [23]. Another advantage is that the multiplexing technology provides a signal feedforward path for the modulator, forming a feedforward modulator structure, which makes the output swing of the integrator independent of the input signal of the modulator, thus improving the overload rate of the modulator. Therefore, the requirements for amplifiers in integrators are greatly reduced, allowing a smaller open-loop gain and lower bandwidth. Since the input signal of the modulator is directly sampled to the capacitor array of the quantizer without going through the integrator, the sum swing of the integrated output is very small, and the analogue adder with a too large swing is not needed, which reduces the design difficulty of the modulator.

Based on the multiplexed SAR quantizer and feedforward technology, the proposed modulator can handle the input signal range close to full amplitude. Therefore, the small capacitance can meet the requirements of circuit thermal noise [24]. The quantizer only needs half of the clock cycle to sample the output of the integrator, and the small C_S_ can greatly reduce the requirements for the second-stage integrator.

## 3. The Overall Circuit Design of the Modulator

**Figure 2:**
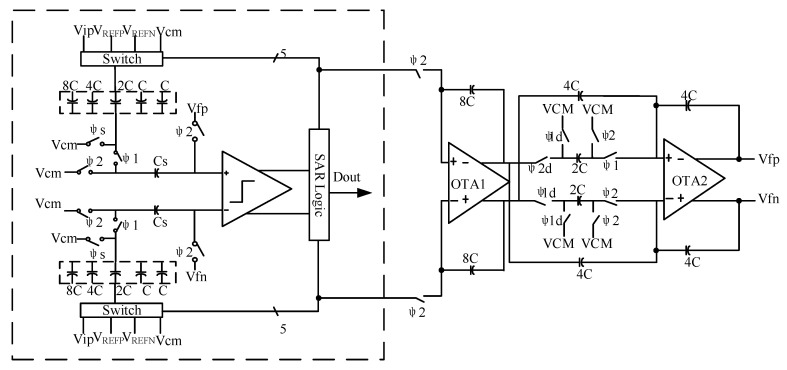
General circuit structure diagram.

**Figure 3:**
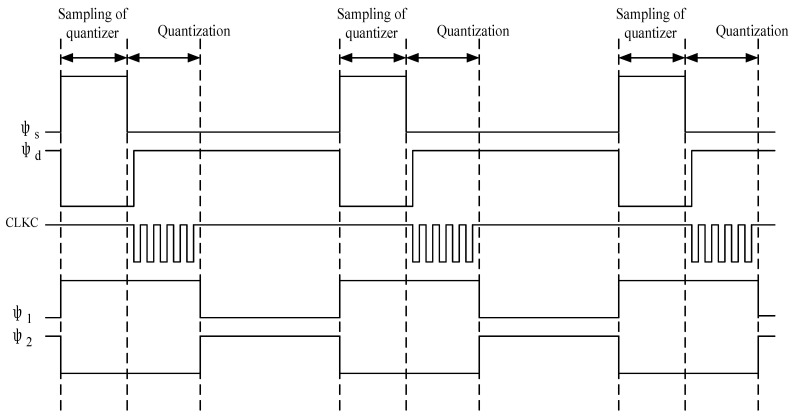
Modulator timing diagram. Where φs is the quantization sampling clock, φd is the quantization conversion clock and Clkc is the comparison clock of the successive approximation register (SAR) comparator [26].

The circuit schematic diagram of the multi-bit modulator is shown in Figure 2. It consists of an integrator, a quantizer, a clock circuit, a capacitor array, a DAC and other units. The main circuit of the modulator is composed of a quantization part and integration part. The clocks in the circuit are the integrator clock and the SAR quantizer clock. The working timing of the whole modulator is shown in Figure 3. In the sampling phase (φ1/φ1d) which is the sampling clock of the integrator, the quantizer is required to complete sampling and digital conversion. In the integration phase (φ2/φ2d) which is the integration clock of the integrator, the quantization result is fed back to the input via the DAC and subtracted from the input signal. CLKC is the control clock of the quantizer comparator, which is generated by the internal circuit of the quantizer. This scheme focuses on the low power consumption design of amplifiers and quantizers [25]. In the imaginary frame of Figure 2, VREFP and VREFN are positive and negative reference voltages, respectively, while VCM is the common-mode voltage, Vin and Vip are input signals and CLKC is the control clock of the comparator. To obtain the required VREFP = 1.8 V, the ADC needs 16 cell capacitors. The clock switch φs is closed in the sampling stage of the quantizer. Meanwhile, φ2 is closed during the whole working period of the quantizer and is turned off when the integrator results are sampled. φ1 is closed when the quantizer samples the integrator result. Vfp and Vfn are the integration results of the integrator feedforward to the positive and negative terminals of the quantizer.

## 4. Quantizer Circuit Module Analysis and Optimization Design

The flash ADC is usually used as a quantizer in multi-bit quantization modulators. The flash ADC requires multiple comparators and also has the problems of a matching circuit and large dynamic power consumption [27]. If a pre-amplifier is added at the front end of the comparator, static power consumption will be increased. In order to address the above-mentioned problems, a scheme of the modulator using a 5-bit SAR ADC as the quantizer is proposed. The SAR ADC has no static power consumption, and the precision is 5 bits, which has no problem of a matching circuit. The sampling frequency of the designed ∆-Σ modulator is 3.2 Ms/s. At this sampling rate, the 5-bit SAR ADC has lower power consumption compared with the flash ADC. In addition, the SAR quantizer structure is used to implement the addition of the adder output signal and the input signal. The adder adds the feedforward signals of the integrator. The output swing of the integrators is very small, which leads to a very small output swing of the adder and therefore greatly reduces the requirements for the amplifier.

The working process of the quantizer is divided into sampling [28] and digital conversion. Both of these two processes are completed in the sampling phase (φ1/φ1d) of the integrator. To avoid using an external high-frequency clock, the comparator clock within the SAR quantizer is generated by the SAR internal circuit, and the specific operation timing is shown in Figure 3. In the sampling phase (φs), the MSB (Most Significant Bit) sampling capacitor is connected to the VREFP, and the lower plate of the other capacitors is connected to the VREPN. The SAR capacitor array samples the output of the integrator while the input signal is sampled to a capacitance equivalent to the entire SAR capacitor array. After the sampling is completed, the sampling switch is turned off to start digital conversion. The capacitor upper plate sampling the input signal is connected to the common-mode VCM, and the SAR quantizer obtains the conversion result through successive comparisons [29]. At the same time as completing the conversion, the SAR quantizer receives the addition of the adder output signal and the input signal. After the digital conversion process is completed, the SAR quantizer outputs the conversion result through control logic.

### 4.1. Sampling Module

Input signal sampling is mainly realized by the gate voltage bootstrap switch circuit, capacitor array and sampling common-mode voltage. Compared with the transmission gate and MOS switch, the gate voltage bootstrap switch is more stable and has lower transmission loss, but the chip area is large, so it is only used in sampling. There is no need for 32 unit capacitors, but only 16 unit capacitors.

**Figure 4:**
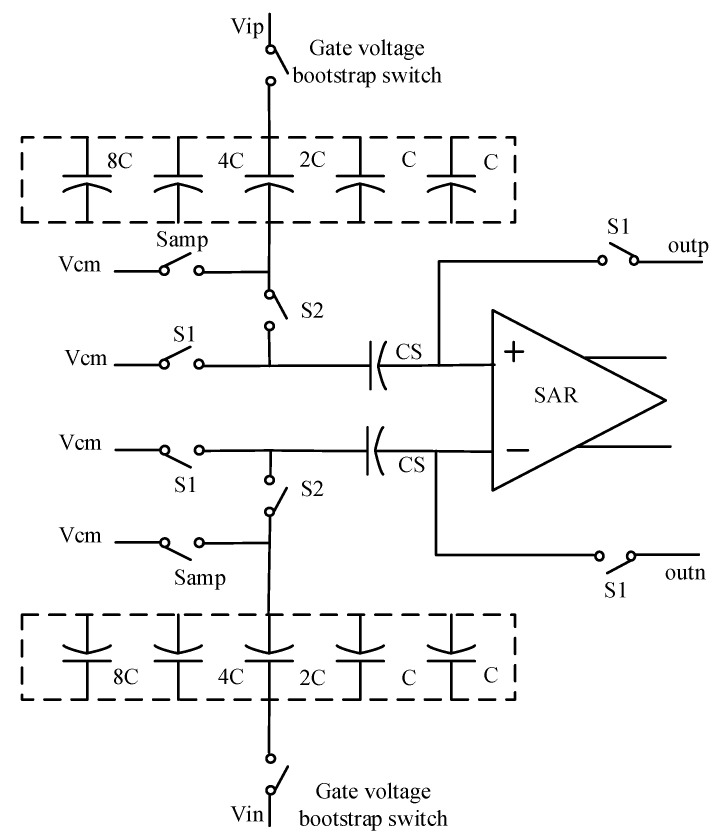
Quantizer sampling circuit.

In the quantizer sampling input signal and conversion stage, S1 is always open and S2 is always closed. Samp and the gate voltage bootstrap switch are switched off after completing sampling. When sampling the output of the integrator, switch S1 is closed and switch S2 is open, the upper plate of the capacitor C_S_ is connected to a common-mode voltage and the lower plate is connected to the integrator output voltages outp and outn. After sampling the integrator, switch S1 is turned off, and the input signal is sampled by the quantizer. Switch S2 is closed, and the voltage value of the upper plate of the capacitor C_S_ becomes the sampled voltage values Vin and Vip, and the lower plate is suspended. By combining the sampling structure of the input signal with the sampling structure of the output of the integrator, the quantizer sampling circuit is formed as shown in Figure 4.

This part mainly optimizes the circuit structure in the following two aspects. First, in order to reduce the power consumption and chip area of the whole circuit, a capacitance multiplexing circuit is adopted. Its structure can reduce the number of capacitors and by choosing a relatively large capacitor size, the capacitor matching gets better. In the second aspect, a proper logic control circuit is adopted in the logic control of the quantizer. As shown in Figure 4, the logic levels of the two points on the left side of the CS capacitor are controlled to maintain a constant common-mode level in the whole quantization process, thus reducing errors caused by inconsistent common-mode levels.

### 4.2. SAR Comparator Module

**Figure 5:**
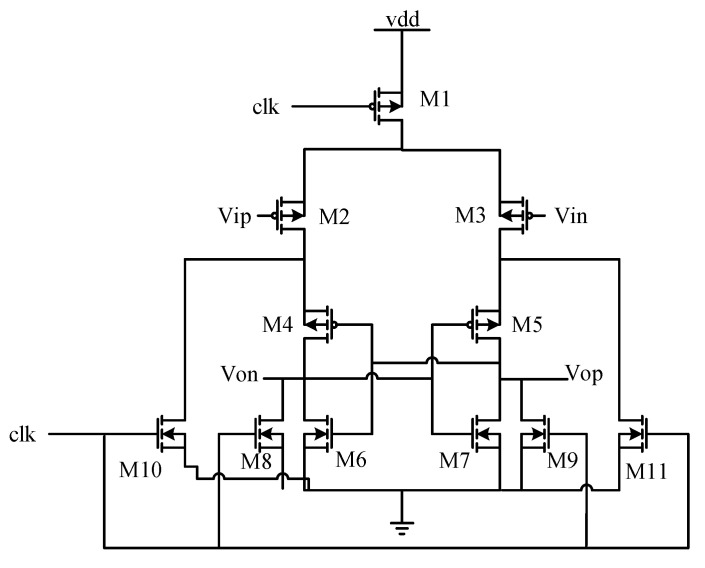
SAR comparator structure.

The SAR comparator adopts a differential structure. It compares the analogue signal obtained by the sampling capacitor with the analogue signal obtained by adding the integrator output and input signal, to finally obtain a digital signal. At the same time, the comparator adopts a dynamic latch structure, which further reduces the power consumption of the system [30]. The latch structure saves the comparison result every time and then resets the comparator state to realize multiplexing. The multiplexing function is completed by the output of the comparator itself cooperating with the external clock circuit. After the output of the comparator is generated, the comparator stops working and resets to prepare for the next comparison. The latch function is realized by the SAR logic control structure, and the quantized values obtained by five comparisons are saved one by one and output in parallel [31]. Compared with the traditional comparator, the SAR comparator adopts a PMOS design and achieves the multiplexing function, and its corresponding schematic diagram is shown in Figure 5.

In Figure 5, Vin and Vip are the positive and negative input ports of the comparator, and Von and Vop are the positive and negative output ports of the comparator. PMOS transistors M2 and M3 are differential input stages connected with input signals. PMOS transistors M4 and M5 have a positive feedback structure, M6 and M7 realize a common source amplification and NMOS transistors M8, M9, M10 and M11 are used for resetting. When the clk signal is at a high level, it is reset. At this time, NMOS transistors M8, M9, M10 and M11 are turned on, and M8 and M9 discharge the output ports, i.e., Von and Vop at the same potential and turn on the PMOS transistors M4 and M5. M10 and M11 make the drain potentials of M2 and M3 which are equal to realize a reset. This can be achieved by comparing M2 and M3 when the clk signal is low which is equivalent to the terminal voltage of Vip greater than the terminal voltage of Vin. At this time, the channel width is opened by M2 which is smaller than M3, and the drain potential of M3 is pulled up quickly, reaching a high level first, so that M4 is turned off and the drain potential of M2 is no longer changed. Then, Vop outputs a high level and Von outputs a low level to complete the comparison [32].

## 5. Integrator Circuit Module Analysis and Optimization Design

Combining the designed transconductance operational amplifier with the switched capacitor, the circuit structure outside the imaginary frame in Figure 2 is obtained. Under the control of the clock switch, the sampling and integration of the integrator are completed. In the figure, φ 2/φ 2d is an integral switch, and φ1/φ1d is a sampling switch, both of which are transmission gates controlled by two clocks that do not overlap each other, in which φ1/φ1d is closed and φ2/φ2d is open. During this period, the quantizer performs sampling and conversion, that is, sampling by the first integrator. Meanwhile, the second-stage integrator samples the results of the first-stage integrator where φ2/φ2d is closed and φ1/φ1d is open. During this period, the quantization result of the quantizer is fed back to the input terminal through the DAC and subtracted from the input signal and sent to the integrator. The first-stage integrator integrates the difference signal, and the second-stage integrator integrates the first-stage result, that is, the output of the integrator is delayed from the input signal by the input signals of two quantizers.

The main components of the integrator are the transconductance operational amplifier and the discrete switched capacitor. Traditional integrators generally adopt a feedback structure, and input signals need to be added before each stage of the integrator input, resulting in a complex circuit structure. In order to further reduce the power consumption of the integrator, a feedforward structure is adopted. This architecture only needs to add the input signal before the first stage input of the integrator and is insensitive to the distortion of the operational amplifier in the integrator. As the swing is small, where the swing of each operational amplifier is about 200 MV, it optimizes the current demand of the amplifier and hence reduces power consumption. Since the SAR ADC is used as the quantizer and the integrator processes the residual difference between the quantization result and the input signal, the first integrator needs no sampling capacitor, which further saves the chip area and hence elaborates the optimization of this part of the circuit.

### 5.1. Design of Transconductance Operational Amplifier Circuit

**Figure 6:**
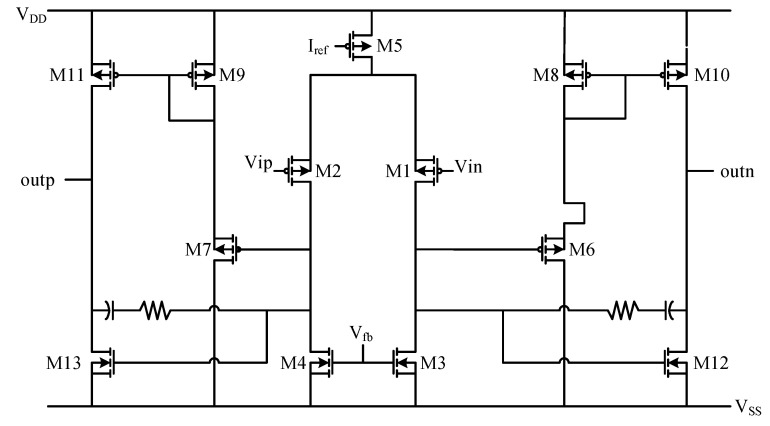
Schematic of transconductance operational amplifier.

According to the principle explanation of the transconductance operational amplifier, the circuit result shown in Figure 6 is designed. In the figure, except for M3, M4, M12 and M13 are NMOS transistors, and the others are PMOS transistors. MOS transistors M1 and M2 are used as differential pairs, M3 and M4 are used as active loads and M5 provides a constant current source to complete the differential input stage. The source follower is composed of M6 and M8, and M7 and M9, and provides a static bias for M10 and M11 at the same time. The VGS of M6 and M7 determines the DC voltage difference between the gates of output stages M10 and M12 and M11 and M13. by adjusting the width/length ratio of M6 and M7, the output offset can be guaranteed to be zero. Resistors and capacitors form a frequency compensation network, which is bridged between the input and output ends of the output amplifier stage.

### 5.2. Design of Reference Voltage Source

**Figure 7:**
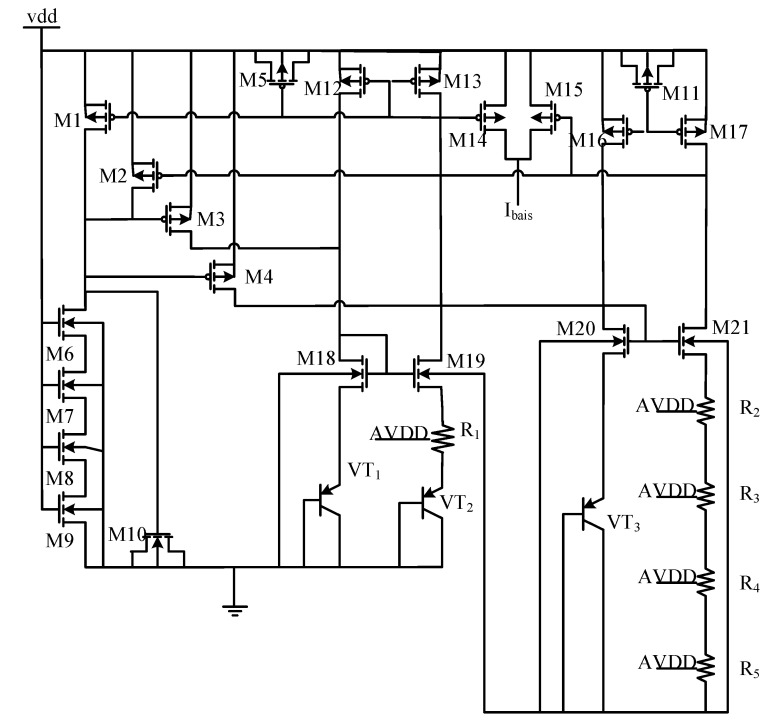
Circuit diagram of the reference voltage source.

Figure 7 is a circuit design diagram of a reference voltage source. As can be noticed, the sources and drains of PMOS transistors M5, M10 and M11 are connected to form a capacitor, which makes the matching design of M1~M4 stabilize the current of the current mirror. M6~M9 and capacitors together stabilize the supply current of M1~M4. PNP transistor VT_2_ is formed by connecting four VT_1_ in parallel to ensure the proportion.

## 6. Pre-Circuit Simulation and Verification

**Figure 8:**
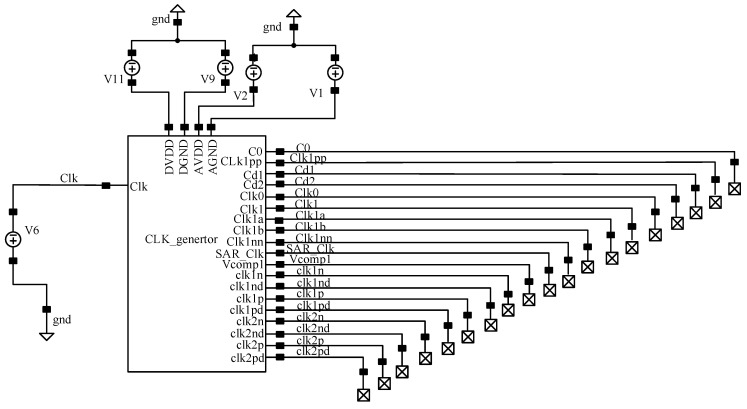
Clock circuit simulation design.

According to the proposed design concept, each module of the modulator is designed separately. In this section, the functional simulation of the designed circuit is carried out by using Cadence spectre simulation software to verify the design scheme and the feasibility of the circuit. The designed clock circuit is integrated and packaged, and the whole clock signal is generated by an external clock, and the clock control timing of the modulator is simulated. The full simulation circuit is shown in Figure 8. As can be seen, the voltage amplitude of CLK is set to 1.8 V, the period is 312.5 ns, the rising and falling times are both 900 ps and the pulse width is 155 ns. The clock signal label in the figure corresponds to the design.

**Figure 9:**
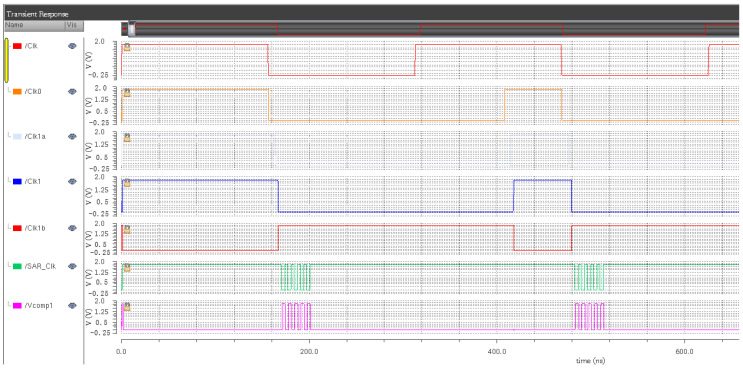
Simulation diagram of the overall clock of the quantization part.

**Figure 10:**
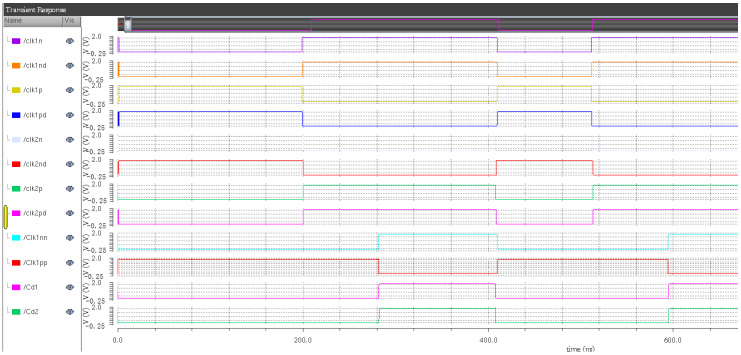
Overall clock simulation diagram of the integral part.

The simulation results can be divided into two parts, the first part is the quantizer sampling clock, quantizer switching clock and circuit switching clock signal, as shown in Figure 9. The second part is the integrator clock, quantizer sampling clock and DAC transmission clock, as depicted in Figure 10. Through the simulation results of the clock circuit, it can be concluded that the clock unit design of each module of the modulator is reasonable, and the clock control signal meets the timing function of each module of the modulator.

**Figure 11:**
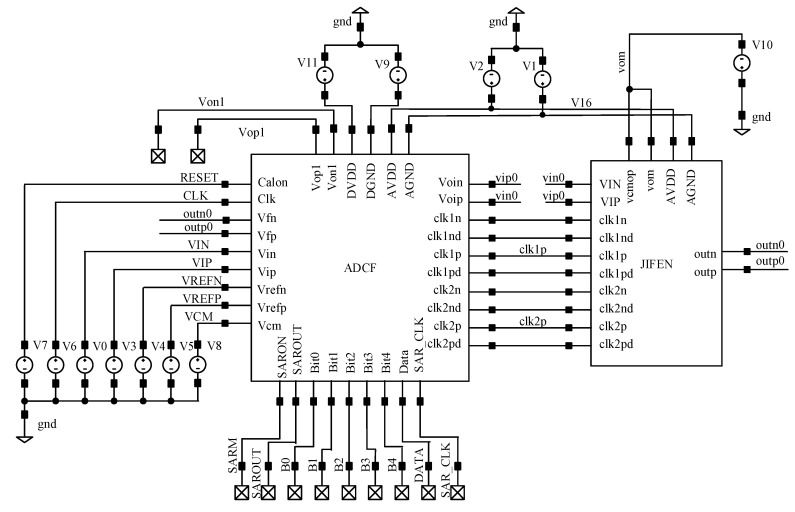
Overall simulation structure diagram of the modulator.

Through the simulation and analysis of the modulator clock unit, the correctness of the overall timing logic of the designed modulator is verified. After each module unit of the circuit is designed, the overall circuit of the modulator circuit is simulated. The simulation circuit is shown in Figure 11, and the signal label in the figure is given in Table 1. Since CLK2p and CLK1p are the integrator integration phase and sampling phase clocks, respectively, only one of them will be explained below. 

**Figure 12:**
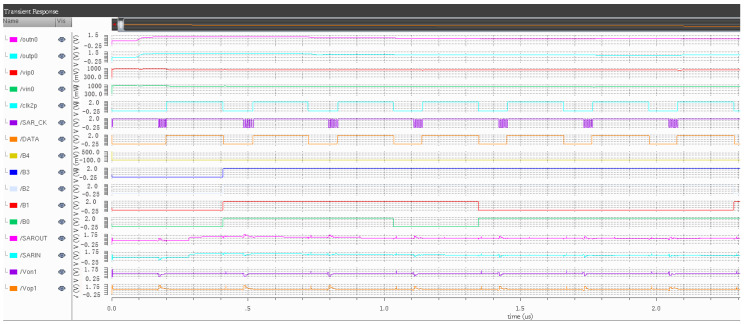
Function simulation diagram of the modulator.

As Cadence spectre takes a long time to simulate the time above microsecond level and cannot simulate the RESET signal in a short time, the RESET is not verified here, so that its grounding has no effect on the circuit. This simulation mainly verifies the logic output of the main signal of the modulator and further confirms the logic function of the modulator. The overall simulation is shown in Figure 12.

By analyzing the simulation results, it can be concluded that after being processed by an integrator, the voltage amplitude of the comparator entering the quantizer is increased to a certain extent, and the voltage applied at both ends of the comparator is appropriately increased, which makes the comparator more stable and also improves the accuracy. The quantizer also works normally under the action of the clock circuit, and the five-bit quantization output of the quantizer and the sampling clock of the quantizer output meet the predetermined output result. The main input/output signals and working clocks of the quantizer and integrator in the simulation diagram correspond to the working principle of the modulator described in the previous section, which verifies the rationality of the proposed working principle of the modulator.

## 7. Layout Design

The layout drawing and verification are completed using a SMIC 180 nm process. A bootstrap switch is used at the input of the ADC to reduce the nonlinearity of on-resistance. The chip makes use of several capacitor units to form capacitors and achieves accurate proportional matching of coefficients. The differential structure of the capacitor array is completely symmetrically distributed on both sides of the comparator, which is used to improve the overall anti-noise capability of the circuit [33]. The digital control logic is uniformly placed at the back end of the chip, and the digital part and the analogue part are effectively isolated to reduce the interference of digital noise on the front-end analogue module. The overall design structure ensures the symmetrical arrangement of analogue parts of the ADC.

**Figure 13:**
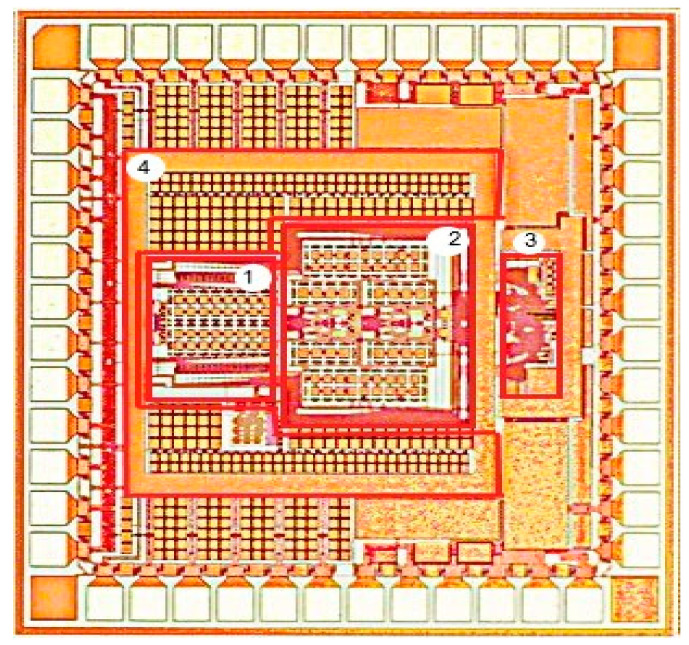
Modulator chip layout, where (1) is the multiplexed sampled capacitor array, (2) is the 2nd-order integrator, (3) is the comparator and (4) is the power supply.

The test chip is fabricated with a 180 nm CMOS process. The design does not require any high-precision capacitance and low threshold voltage process. Figure 13 shows a micrograph of the chip. The overall chip area is 1360 × 1360 µm^2^ and the core area is 966 × 748 µm^2^.

## 8. Chip Testing and Comparison of Previous Works

The performance comparison of the presented work with other published works can be found in Table 2. As can be seen, the designed modulator chip has lower power consumption and better performance against others. By measuring the voltage and current on the PCB of the chip, the test power consumption of this design chip is found to be 3.654 mW. However, there are some losses from the auxiliary devices and power supply on the testing board, which result from the high test data. If other device losses and power losses are not considered, the actual power consumption of the chip is less than 2.5 mW. Reference [18] adopts a second-order 4-bit quantizer structure which has a simulated power consumption of 69 μW and lower than the proposed architecture. However, it adopts a 65 nm process and the simulated data does not consider the power and circuit loss, so it cannot be compared at the same level. Meanwhile, the SNDR value of the proposed architecture is 106 dB which demonstrates the obvious advantages of the proposed architecture.

**Figure 14:**
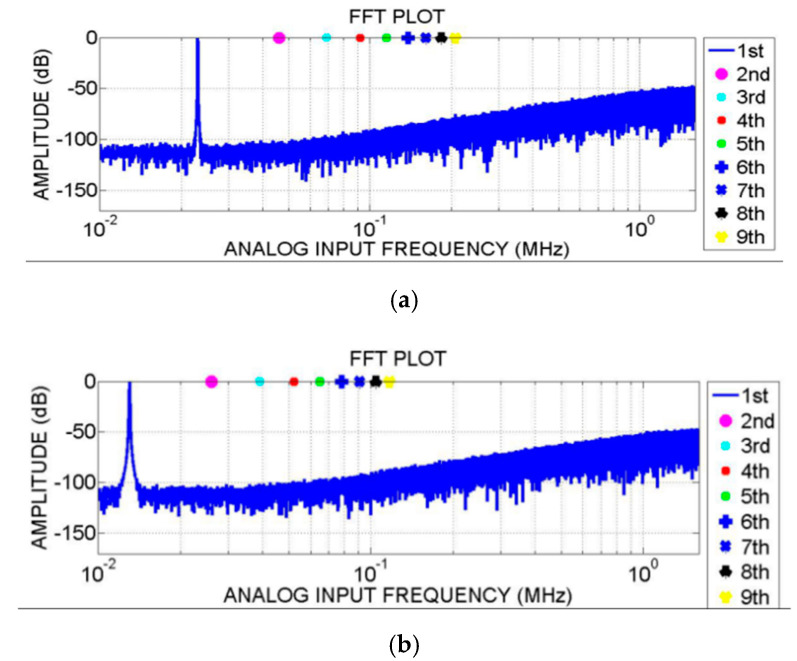

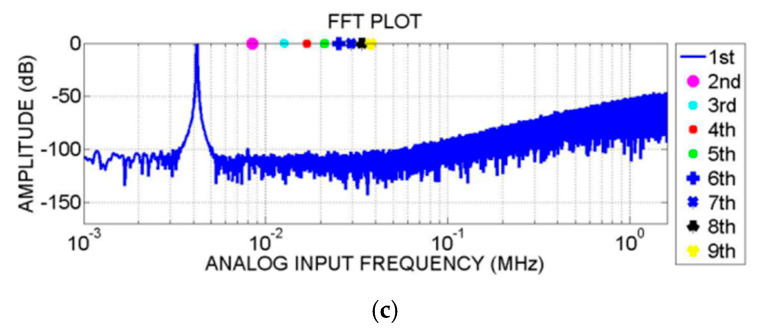
Output power spectrum of the modulator at different input frequencies. (**a**) Spectrum output of the modulator at 23 K input frequency. (**b**) Spectrum output of the modulator at 13 K input frequency. (**c**) Spectrum output of the modulator at 5.1 K input frequency.

By using a 3.2 MS/s sampling rate, the output power spectrums with the oversampling ratio of 128 of the delta-sigma ADC at three selected differential input sine waves, i.e., 5.1, 12 and 23 K, are shown in Figure 14. In the current architecture, when the signal bandwidth (25 K) is fixed, the oversampling ratio (OSR) is determined by the expected precision of the modulator and the highest sampling rate of the sampling circuit. For quantization noise, the higher the oversampling ratio is, the higher the signal-to-noise ratio (SNR) that can be achieved by the modulator. However, the final accuracy of the modulator depends on the upper limit of the accuracy that can be realized by the sampling circuit, thus limiting the sampling rate of the modulator and ultimately affecting the oversampling ratio of the modulator.

**Figure 15:**
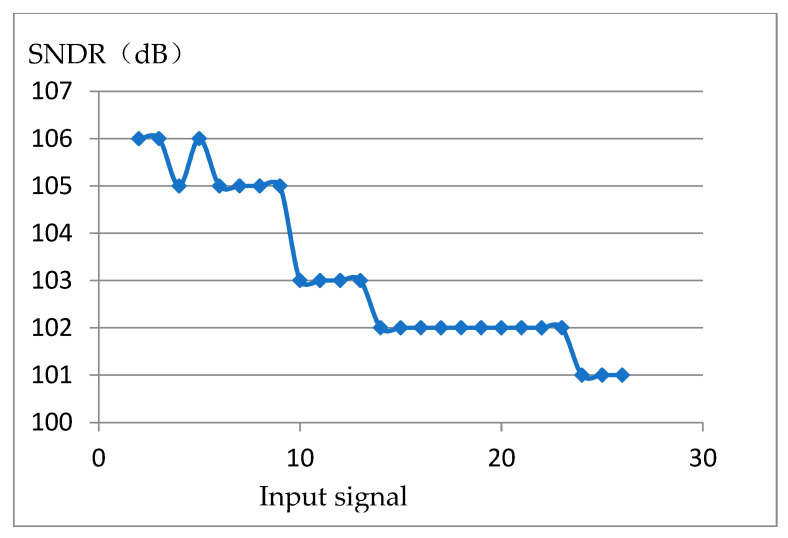
Measured relationship between input signal frequency and signal to noise distortion ratio (SNDR).

The results confirmed the consistent performance of the output power spectrums across the desired operating input sine wave frequency band from 0 to 25 KHz. To evaluate the corresponding SNDR performance, Figure 15 illustrates the measured SNDR against the input signal frequency. It can be seen that the peak SNDR is 106 dB at 3 kHz and the lowest SNDR is 101 dB at 25 KHz. Figure 15 shows the SNDR point connection diagram of different integer signal frequencies under the same signal amplitude in the actual test. As can be seen, SNDR decreases with the increase in the signal frequency due to the presence of clock jitter and nonlinear factors. Data weighted averaging (DWA) is used in the feedback capacitor array, which reduces the harmonic components such as a second harmonic and third harmonic caused by capacitor array mismatch, thus improving the overall dynamic range. The test results show that the dynamic range can exceed 100 dB. The total power consumption of the chip is 3.654 mW. The supply voltage of both analogue and digital circuits is 1.8 V and the Figure of Merit (FOM) is 169.4 dB.

## 9. Conclusions

Employing a second-order 5-bit quantization structure, a Σ-Δ modulator with low power consumption and a high-resolution modulator scheme was proposed for analogue-to-digital converters. Through optimizing the circuit structure, a reusable quantizer sigma-delta modulator based on the SAR structure was presented. Under the condition of a 128 oversampling rate, it demonstrates a resolution of 16 bits, power consumption of 3.654 mW and FOMs of 169.4 dB for 0 to 25 kHz analogue signals, showing it has higher data conversion efficiency and a higher optimal value. These results confirmed that the modulator of this structure meets the requirements of low power consumption and high precision for audio applications. The whole modulator was realized by a SMIC single-layer polysilicon six-layer metal 180 nm CMOS process where the working power supply voltage was 1.8 V. In comparison with other reported modulators realized by a 180 nm CMOS process, the proposed architecture offers several advantages and is far superior retrospectively with lower power consumption.

## Figures and Tables

**Table 1:** Description table of the modulator structure diagram.

Name	Representative Significance	Name	Representative Significance
RESET	Modulator reset signal	CLK	Total clock input signal
outn0	Negative feedforward of integrator	VREFP	Voltage reference
outp0	Integrator forward feed	VCM	Common-mode voltage
VIN	Negative input signal	VREFN	Terminal voltage at ground
VIP	Positive input signal	Von1	DAC negative feedback
B0~B4	Five-bit quantization	Vop1	DAC positive feedback
DATA	Quantization result output clock	vip0	Negative input of integrator
SARIN	Comparator negative signal	vin0	Positive input of integrator
SAROUT	Positive signal of comparator	clk2p	Integrator integrated phase clock

**Table 2:** Parameter comparison.

	Power Consumption (mW)	Frequency (MHz)	FOMs (dB)	CMOS Technology (μm)	SNDR (dB)	OSR	AREA (mm^2^)
[34]	14.7	8.0	172.2	0.18	105.9	128	-
[35]	8.1	-	-	0.18	81.0	-	-
[36]	6.65	960	150.7	0.028	-	48	0.015625
[37]	12.7	0.64	165.0	0.35	-	320	11.48
[38]	475	2.5	-	0.25	100.0	-	-
[39]	18.5	-	-	0.65	72.3	-	0.25
[40]	5	256	160.4	0.13	74.4	64	0.33
[41]	16	600	166.0	0.090	78	30	0.36
[42]	3.2	1	165.6	0.35	100.2	250	3.8
This work	3.65	3.2	169.4	0.18	106	128	0.56
